# Design, Synthesis and Evaluation of Indene Derivatives as Retinoic Acid Receptor α Agonists

**DOI:** 10.3390/molecules22010032

**Published:** 2016-12-27

**Authors:** Xianghong Guan, Peihua Luo, Qiaojun He, Yongzhou Hu, Huazhou Ying

**Affiliations:** ZJU-ENS Joint Laboratory of Medicinal Chemistry, College of Pharmaceutical Sciences, Zhejiang University, Hangzhou 310058, China; guanx082@umn.edu (X.G.); peihualuo@zju.edu.cn (P.L.); qiaojunhe@zju.edu.cn (Q.H.); huyz@zju.edu.cn (Y.H.)

**Keywords:** retinoic acid receptor, agonist, all-*trans*-retinoic acid derivative, indene, structure and activity relationship

## Abstract

A series of novel indene-derived retinoic acid receptor α (RARα) agonists have been designed and synthesized. The use of receptor binding, cell proliferation and cell differentiation assays demonstrated that most of these compounds exhibited moderate RARα binding activity and potent antiproliferative activity. In particular, 4-((3-isopropoxy-2,3-dihydro-1*H*-inden-5-yl)-carbamoyl)benzoic acid (**36d**), which showed a moderate binding affinity, exhibited a great potential to induce the differentiation of NB4 cells (68.88% at 5 μM). Importantly, our work established indene as a promising skeleton for the development of novel RARα agonists.

## 1. Introduction

**Figure 1:**
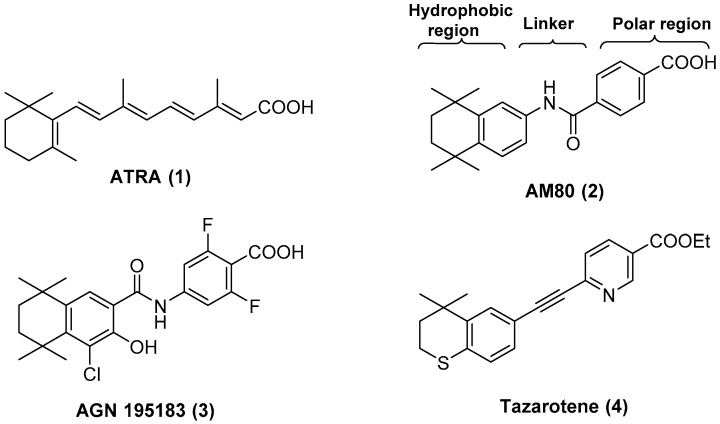
Chemical structures of ATRA (**1**) and representative aromatic RA derivatives (**2**–**4**).

The retinoid signal is mediated in target cells through retinoic acid receptors (RAR) and retinoid X receptors (RXR), both of which are members of the nuclear receptor superfamily. RARs are ligand-dependent transcription factors that act as RAR-RXR heterodimers to modulate gene transcription and thereby regulate a range of metabolic, endocrine and immunologic disorders [1,2]. There are three distinct isoforms RAR (α, -β and -γ), among which RARα is known to play a pivotal role in the control of cellular differentiation and apoptosis, and is therefore an important drug target for cancer therapy and prevention [3]. The natural ligand of RARα, all-*trans*-retinoic acid (ATRA), has been used to effectively treat acute promyelocytic leukaemia (APL) for nearly thirty years [4]. However, this therapy has its limitations which mainly lie in the structure of ATRA. Due to the presence of conjugated double bonds, ATRA easily undergoes oxidation and/or isomerization in the presence of oxidants, light or excessive heat [5]. To improve the stability, a large number of derivatives have been developed by fusing an aromatic ring in both its hydrophobic and hydrophilic regions to constrain the polyene side chain. The study of the relationships between structure and activity (SAR) has established the structure template of ATRA derivatives as a hydrophobic region and a polar region connected via a linker (Figure 1) [6,7,8]. Further SAR has revealed that the nature of the linker is crucial for the compounds to attain RAR-isotype selectivity and that the amide linker group is a key structural feature for RARα-specificity, presumably due to a favorable hydrogen-bonding interaction between the amide group of the ligand and the hydroxyl group of serine 232 residue present in the ligand binding pocket of RARα [9,10].

Among all the ATRA derivatives, AM80 (Figure 1) is a typical representative approved for therapy in 2005. AM80 can successfully induce complete remission in APL patients for whom ATRA therapy has failed [11,12,13]. This implies the great potential of synthetic RARα agonists in the treatment of APL and has fostered the search for new classes of compounds with improved pharmacologic activities.

**Figure 2:**
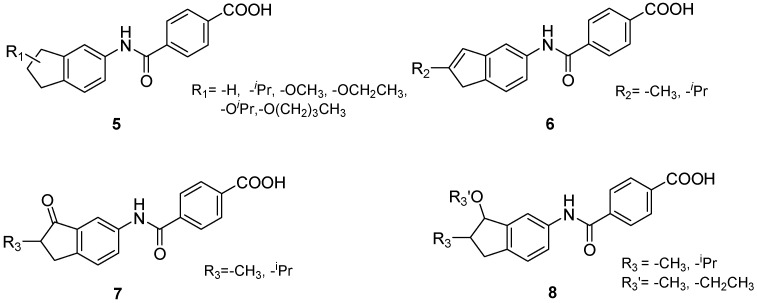
Chemical structures of indene derived compound series **6**–**8**.

To date, most of the developed ATRA derivatives contain a tri-/tetra-methylated six-membered rigid ring in their hydrophobic region. Since it has been reported that the size of the hydrophobic part of the ligands can significantly affect the activity [10], we were interested in studying the impact of a smaller ring system on the activity of derivatives by replacing the hydrophobic part of AM80 with mono-/di- substituted indene derivatives. Specifically, compound **5** was designed by incorporating small alkoxyl or alkyl groups into the indane structure. Keeping the planar configuration of indene by retaining the double bond or the incorporation of a ketone group yielded compounds **6** and **7**. Di-substitution of indane with both alkoxyl and alkyl groups gave compounds **8** (Figure 2).

## 2. Chemistry

**Scheme 1:**
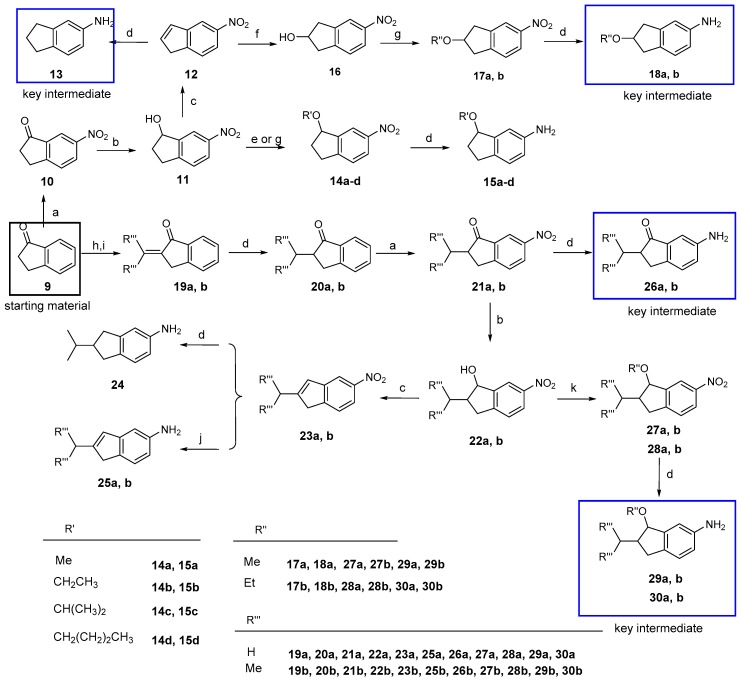
The synthetic route of **13**, **18a**, **18b**, **26a**, **26b**, **29a**, **29b**, **30a**, **30b**. *Reagents and conditions*: (a) KNO_3_, H_2_SO_4_, 0 °C, 4 h; (b) NaBH_4_, MeOH/THF = 2/1, 1 h; (c) *p*-toluenesulfonamide, PhMe, reflux, 2 h; (d) H_2_, 10% Pd/C, EtOAc, r.t., overnight; (e) MeI or EtI, MeONa, THF, r.t., 12 h; (f) (i) diborane, THF, r.t., 2 h; (ii) 30% H_2_O_2_, 30% KOH aq, r.t., 1 h; (g) 2-bromo-propane or 1-bromobutane HgO/HBF_4_, CH_2_Cl_2_, r.t., 2 h; (h) paraformaldehyde, AcOH, morpholine, reflux, 2 h; (i) acetone, NaOH, r.t., 4 h; (j) Fe, AcOH, EtOH, reflux, 2 h; (k) methyl orthoformate or triethoxy orthoformate, BiCl_3_, CH_2_Cl_2_, r.t., 7 h.

Synthesis of indene derivatives **36a**–**p** was conducted via procedures reported for the preparation of AM80 with some modifications. Detailed syntheses are shown in Scheme 1. Nitration of commercial available **9** with KNO_3_ and H_2_SO_4_ gave **10**, whose carbonyl group was then reduced by NaBH_4_ to yield **11**. Elimination of H_2_O from **11** gave **12**, which could either be reduced to **13** or transformed into **16** [14,15]. Etherification of **16** with alkyl halides in the presence of KOH produced **17a** and **17b**. Compound **11** could also be alkylated with appropriate halogenoalkane (MeI, EtI, 2-bromo-propane or 1-bromobutane) to afford **14a**–**d**. In another synthetic route, **9** was reacted with paraformaldehyde/acetone to give **19a** and **19b**, which were reduced to yield **20a** and **20b**. Subsequent nitration of **20a** and **20b** and reduction of the carbonyl group gave **22a** and **22b**. Compounds **23a** and **23b** could be obtained by elimination reaction of **22a** and **22b**, while coupling of **22a**, **22b** with trimethyl orthoformate or triethyl orthoformate in the presence of BiCl_3_ yielded **27a**, **28a** and **27b**, **28b**, respectively. Reduction of the nitro group in **14a**–**d**, **17a**, **17b**, **21a**, **21b**, **23a**, **23b**, **27a**, **27b** and **28a**, **28b** then yielded the hydrophobic moieties **15a**–**d**, **18a**, **18b**, **26a**, **26b**, **24**, **25a**, **25b**, **29a**, **29b** and **30a**, **30b** [16].

**Scheme 2:**
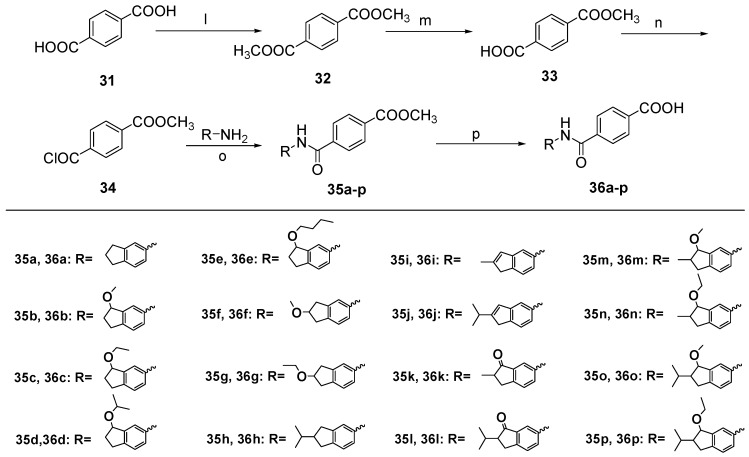
The synthetic route of **36a**–**36p**. *Reagents and conditions*: (l) MeOH, SOCl_2_, r.t., 12 h; (m) KOH, MeOH, ether, H_2_O, r.t., 24 h; (n) SOCl_2_, reflux, 24 h; (o) amine fragments, Py, CH_2_Cl_2_, r.t., 7 h; (p) 0.5% LiOH aq, MeOH, r.t., 48 h.

The hydrophilic segment **34** was prepared from terephthalic acid (**31**) after esterification and hydrolysis, followed by chlorination. Coupling of **34** with the hydrophobic part yielded **35a**–**p** which were then hydrolyzed to give the target ligands **36a**–**p** (Scheme 2).

## 3. Results and Discussion

### 3.1. RARα Binding Affinity

The obtained target compounds were tested for their binding affinities to RARα using a time resolved fluorescence resonance energy transfer (TR-FRET) assay with AM80 as the positive control. As shown in Table 1, compound **36a** which bears no substituents exhibits modest RARα binding affinity, implying the feasibility of the indene skeleton as a promising platform for novel RARα agonists. With **36b**–**36g** being less potent than **36a**, it seems that an alkoxyl group is not welcome, especially at the 2-position.

Furthermore, the extension of the π system by the retention of the indene double bond or the incorporation of a ketone group at the 1-position seems to be favorable, with **36j** and **36l** being more potent than their more saturated counterpart **36h**. Interestingly, although an alkoxy-substituent alone is not well tolerated, it contributes to the binding affinity when coexisting with an isopropyl group, which is illustrated by comparison of the results of **36o**, **36p** with those of **36b** and **36c**.

### 3.2. Cell Proliferation Inhibitory Assay

Human promyelocytic leukemia cell lines HL60 and NB4 were employed to determine the effects of the derivatives on cell proliferation [17]. As shown in Table 2, these two cell lines responded quite differently to the tested compounds. Compounds **36b**–**c** with small aliphatic ether chains at the 1-position are more potent than **36d**–**e** with larger ether side chains in HL60 cells, while the results in NB4 cells are the contrary. Alkoxy-substitution at the 2-position (i.e., compounds **36f**, **36g**) harmed the cell proliferation inhibitory activity of the compounds in both cell lines while the isopropyl group at the same position (compound **36h**) is favourable. The extension of the π system by an alkenyl bond or a ketone group, as in **36j**–**l**, didn’t affect the compounds’ inhibitory activity in HL60 cells but nulified their activity in NB4 cells. The indene ring tolerates the dual-introduction of a small alkoxyl and an alkyl group at 1- and 2-position, respectively, to retain the antiproliferative activity in HL60 cells. The results of the dual-substituted compounds in the NB4 cells line are a little complicated, with **36n** and **36p** bearing an ethoxyl group at the 1-position showing moderate activity and **36m** and **36o** with a methoxyl group at the same position being almost inactive.

### 3.3. Cell Differentiation Assay Using HL60 and NB4

The effects of **36a**–**g** on the differentiation of HL60 and NB4 cells were then assessed. FACS analysis of the granulocyte differentiation marker CD11b revealed that **36d** and **36e**, which show high binding affinity to RARα (14.88~24.25 nM) and potent proliferation inhibitory activity in NB4 cells (1.86~4.09 μM), have the greatest potential to induce NB4 cell maturation (Table 3), which is in correspondence with the molecular basis of APL [18].

## 4. Materials and Methods

### 4.1. General Information

Unless otherwise noted, all reagents were purchased from commercial suppliers and used without further purification. Anhydrous THF was distilled from Na prior to use. Reactions were monitored by thin layer chromatography using TLC Silica gel 60 F_254_ supplied by Qingdao Puke Separation Material Corporation (Qingdao, China). Silica gel for column chromatography was 200–300 mesh and was supplied by Qingdao Marine Chemical Factory (Qingdao, China). Characterization of intermediates and final compounds was done using NMR spectroscopy and mass spectrometry. ^1^H-NMR spectra (500 MHz) were determined in CDCl_3_ on an Advance III MHz spectrometer (Bruker, Bremen, Germany) with TMS as internal standard. Chemical shifts are expressed in parts per million (ppm) and coupling constants in Hz. Mass spectra (ESI-MS) were recorded on an Esquire-LC-00075 spectrometer (Bruker, Bremen, Germany). HRMS were recorded on a 6224 TOF LC/MS spectrometer (Agilent, Santa Clara, CA, USA). Purity was confirmed on a Agilent 1100 series HPLC system equipped with a C18 column (Eclipse XDB-C18, 5 μm, 4.6 × 250 mm) eluted in gradient mode with CH_3_CN in H_2_O (from 10% to 95%). Melting points were measured with a B-540 melting-point apparatus (Büchi, Flawil, St. Gallen, Switzerland) and are uncorrected. 

### 4.2. Chemistry

*6-Nitro-2,3-dihydro-1H-inden-1-one* (**10**). To a solution of 2,3-dihydro-1*H*-inden-1-one (**9**, 1.32 g, 10.0 mmol) in concentrated sulfuric acid (10 mL), KNO_3_ (1.21 g, 12.0 mmol) in concentrated sulfuric acid (10 mL) was added dropwise at −5 °C in 30 min. The mixture was stirred at −5 °C for 4 h. After adding ice water slowly, the mixture was partitioned between water and CH_2_Cl_2_. The organic layer was washed with a saturated aqueous solution of NaHCO_3_ and brine, dried over anhydrous Na_2_SO_4_, and concentrated under vacuum. The residue was purified by column chromatography on silica gel (eluent: hexane/EtOAc = 7/1) to give **10** (1.10 g, 62%) as a beige solid. m.p. 71~74 °C; ^1^H-NMR: δ 8.59 (d, *J* = 1.9 Hz, 1H), 8.47 (dd, *J* = 8.4, 2.2 Hz, 1H), 7.68 (t, *J* = 8.4 Hz, 1H), 3.33–3.25 (m, 2H), 2.89–2.78 (m, 2H); ESI-MS: *m*/*z* [M + H]^+^ 178.

*6-Nitro-2,3-dihydro-1H-inden-1-ol* (**11**). To a solution of **10** (1.77 g, 10.0 mmol) in a mixed solution of MeOH/THF (2:1, 20 mL), NaBH_4_ (1.52 g, 40.0 mmol) was added in portions. The mixture was stirred at room temperature for one hour. After the addition of water (40 mL), the mixture was partitioned between water and EtOAc. The organic layer was washed with a saturated aqueous brine, dried over anhydrous Na_2_SO_4_, and concentrated under vacuum. The residue was purified by column chromatography on silica gel (eluent: hexane/EtOAc = 2/1) to give **11** (1.64 g, 92%) as a white solid. m.p. 74~77 °C; ^1^H-NMR: δ 8.26 (d, *J* = 1.8 Hz, 1H), 8.15 (dd, *J* = 8.3, 2.1 Hz, 1H), 7.38 (d, *J* = 8.3 Hz, 1H), 5.32 (t, *J* = 6.2 Hz, 1H), 3.14 (m, 1H), 2.98–2.85 (m, 1H), 2.64–2.58 (m, 1H), 2.09–1.97 (m, 1H); ESI-MS: *m*/*z* [M + H]^+^ 180.

*5-Nitro-1H-indene* (**12**). To a solution of **11** (1.79 g, 10.0 mmol) in anhydrous toluene (20 mL), TsOH (1.72 g, 10.0 mmol) was added at room temperature and the mixture was refluxed for 2 h. The solvent was removed by distillation. After adding water (40 mL), the mixture was partitioned between water and EtOAc. The organic layer was washed with a saturated aqueous solution of NaHCO_3_ and brine, dried over anhydrous Na_2_SO_4_, and concentrated under vacuum. The residue was purified by column chromatography on silica gel (eluent: hexane/EtOAc = 10/1) to give **12** (1.38 g, 86%) as a white solid, m.p. 78~81 °C; ^1^H-NMR: δ 8.23 (d, *J* = 2.1 Hz, 1H), 8.11 (dd, *J* = 8.2, 2.1 Hz, 1H), 7.58 (d, *J* = 8.2 Hz, 1H), 6.96 (d, *J* = 5.5 Hz, 1H), 6.76 (dt, *J* = 5.4, 1.9 Hz, 1H), 3.52 (s, 2H);ESI-MS: *m*/*z* [M + H]^+^ 162.

*1-Methoxy-6-nitro-2,3-dihydro-1H-indene* (**14a**). To a solution of **11** (90 mg, 0.5 mmol) and CH_3_I (0.31 mL, 5.0 mmol) in anhydrous THF (2 mL), CH_3_ONa (108 mg, 2.0 mmol) was added at 0 °C. The mixture was stirred at room temperature for 12 h. After adding water (20 mL), the mixture was partitioned between water and EtOAc. The organic layer was washed with saturated brine, dried over anhydrous Na_2_SO_4_, and concentrated under vacuum. The residue was purified by column chromatography on silica gel (eluent: hexane/EtOAc = 30/1) to give **14a** (39 mg, 41%) as a pale yellow liquid. ^1^H-NMR: δ 8.26 (d, *J* = 2.0 Hz, 1H), 8.17 (dd, *J* = 8.3, 2.2 Hz, 1H), 7.40 (d, *J* = 8.3 Hz, 1H), 4.87 (dd, *J* = 6.5, 4.5 Hz, 1H), 3.46 (s, 3H), 3.20–3.13 (m, 1H), 2.95–2.89 (m, 1H), 2.52–2.40 (m, 1H), 2.25–2.13 (m, 1H); ESI-MS: *m*/*z* [M + H]^+^ 194.

*1-Ethoxy-6-nitro-2,3-dihydro-1H-indene* (**14b**). The title compound was prepared (34 mg, 33%) as a pale yellow liquid from **11** and CH_3_CH_2_I in a similar method with that described for **14a**. ^1^H-NMR: δ 8.24 (d, *J* = 2.1 Hz, 1H), 8.14 (dd, *J* = 8.3, 2.2 Hz, 1H), 7.37 (d, *J* = 8.3 Hz, 1H), 4.95 (q, *J* = 6.5 Hz, 1H), 3.70–3.60 (m, 2H), 3.18–3.12 (m, 1H), 2.93–2.84 (m, 1H), 2.50–2.43 (m, 1H), 2.19–2.12 (m, 1H), 1.27 (t, *J* = 7.0 Hz, 3H). ESI-MS: *m*/*z* [M + H]^+^ 208.

*1-Isopropoxy-6-nitro-2,3-dihydro-1H-indene* (**14c**). To a stirred solution of compound **11** (180 mg, 1.0 mmol) and isopropyl bromide (183 mg, 1.5 mmol) in anhydrous CH_2_Cl_2_ (2 mL), dry mercury oxide/tetrafluoroboric acid (190 mg, 0.5 mmol) was added. The mixture was stirred at room temperature for 2 h and then treated successively with NaHCO_3_ and 3 M potassium hydroxide until basic. The precipitated mercury oxide was filtered off and the filtrate was extracted with CH_2_Cl_2_. The organic layer was washed with brine, dried over anhydrous Na_2_SO_4_, and concentrated under vacuum. The residue was purified by column chromatography on silica gel (eluent: hexane/EtOAc = 25/1) to give **14c** (42 mg, 19%) as a pale yellow liquid. ^1^H-NMR: δ 8.19 (d, *J* = 2.0 Hz, 1H), 8.12 (dd, *J* = 8.3, 2.2 Hz, 1H), 7.35 (d, *J* = 8.3 Hz, 1H), 5.03 (t, *J* = 6.3 Hz, 1H), 3.93–3.84 (m, 1H), 3.15–3.09 (m, 1H), 2.94–2.82 (m, 1H), 2.54–2.48 (m, 1H), 2.12–2.05 (m, 1H), 1.29 (d, *J* = 6.1 Hz, 3H), 1.26 (d, *J* = 6.1 Hz, 3H). ESI-MS: *m*/*z* [M + H]^+^ 222.

*1-Butoxy-6-nitro-2,3-dihydro-1H-indene* (**14d**). The title compound was prepared as a pale yellow liquid (69 mg, 29%) from **11** and 1-bromobutane in a manner similar to that described for **14c**. ^1^H-NMR: δ 8.22 (s, 1H), 8.13 (d, *J* = 8.2 Hz, 1H), 7.36 (d, *J* = 8.2 Hz, 1H), 4.93 (t, *J* = 5.5 Hz, 1H), 3.69–3.50 (m, 2H), 3.20–3.07 (m, 1H), 2.97–2.83 (m, 1H), 2.55–2.42 (m, 1H), 2.22–2.02 (m, 1H), 1.70–1.56 (m, 2H), 1.41 (m, 2H), 0.94 (t, *J* = 6.4 Hz, 3H);ESI-MS: *m*/*z* [M + H]^+^ 236.

*5-Nitro-2,3-dihydro-1H-inden-2-ol* (**16**). To a stirred solution of compound **12** (805 mg, 5.0 mmol) in anhydrous THF, diborane (10 mmol) in diethyl sulfide (5 mL) was added dropwise at 0 °C. The mixture was stirred at room temperature for 2 h. A small amount of water was added until no bubbles were generated, then 30% hydrogen peroxide (2.8 mL) was added followed by the addition of 1 N NaOH (0.6 mL). The mixture was stirred at room temperature for another 1 h. After adding water (50 mL), the mixture was partitioned between water and EtOAc. The organic layer was washed with a saturated aqueous solution of brine, dried over anhydrous Na_2_SO_4_, and concentrated under vacuum. The residue was purified by column chromatography on silica gel (eluent: hexane/EtOAc = 3/1) to give **16** (295 mg, 33%) as a white solid. m.p. 90~92 °C; ^1^H-NMR: δ 8.10 (s, 1H), 8.07 (d, *J* = 8.2 Hz, 1H), 7.37 (d, *J* = 8.2 Hz, 1H), 4.85–4.77 (m, 1H), 3.34–3.24 (m, 2H), 3.01 (m, 2H); ESI-MS: *m*/*z* [M + H]^+^ 180.

*2-Methoxy-5-nitro-2,3-dihydro-1H-indene* (**17a**). The title compound was prepared from **16** and iodomethane in a manner similar to that described for **14a** as a pale yellow liquid (41%). ^1^H-NMR: δ 8.08 (s, 1H), 8.04(d, *J* = 8.0 Hz, 1H), 7.35 (d, *J* = 8.2 Hz, 1H), 4.33–4.29 (m, 1H), 3.38 (s, 3H), 3.24–3.19 (m, 2H), 3.11–3.06 (m, 2H);ESI-MS: *m*/*z* [M + H]^+^ 194.

*2-Ethoxy-5-nitro-2,3-dihydro-1H-indene* (**17b**). The title compound was prepared from **16** and iodoethane in a manner similar to that described for **14a** as a pale yellow liquid (26%). ^1^H-NMR: δ 7.97 (d, *J* = 11.5 Hz, 2H), 7.27 (s, 1H), 4.37–4.30 (m, 1H), 3.48 (q, *J* = 7.0 Hz, 2H), 3.15 (dd, *J* = 17.0, 6.3 Hz, 2H), 2.99 (dt, *J* = 9.1, 4.3 Hz, 2H), 1.14 (t, *J* = 7.0 Hz, 3H);ESI-MS: *m*/*z* [M + H]^+^ 208.

*2-Methylene-2,3-dihydro-1H-inden-1-one* (**19a**). To a solution of **9** (1.32 g, 10.0 mmol) and paraformaldehyde (1.50 g, 5.0 eq) in glacial acetic acid (20 mL), morpholine (0.5 mL) was added. The mixture was refluxed under nitrogen for 2 h. The glacial acetic acid was removed by distillation. After adding water (50 mL), the mixture was partitioned between water and EtOAc. The organic layer was washed with brine, dried over anhydrous Na_2_SO_4_, and concentrated under vacuum. The residue was purified by column chromatography on silica gel (eluent: hexane/EtOAc = 7/1) to give **19a** (0.45 g, 31%) to give a yellow liquid. ^1^H-NMR: δ 7.89 (d, *J* = 7.6 Hz, 1H), 7.62 (t, *J* = 7.4 Hz, 1H), 7.51 (d, *J* = 7.6 Hz, 1H), 7.42 (t, *J* = 7.4 Hz, 1H), 6.39 (s, 1H), 5.65 (s, 1H), 3.78 (s, 2H); ESI-MS: *m*/*z* [M + H]^+^ 145.

*2-(Propan-2-ylidene)-2,3-dihydro-1H-inden-1-one* (**19b**). To a solution of **9** (1.32 g, 10.0 mmol) in anhydrous acetone (20 mL), NaOH (132 mg, 3.3 mmol) was added at room temperature. The mixture was stirred at room temperature for 4 h and then neutralized with 1 N HCl. Acetone was removed by distillation. After adding water (50 mL), the mixture was partitioned between water and EtOAc. The organic layer was washed with brine, dried over anhydrous Na_2_SO_4_, and concentrated under vacuum. The residue was purified by column chromatography on silica gel (eluent: hexane/EtOAc = 7/1) to give **19b** (0.77 g, 45%) as a yellow solid. m.p. 98~100 °C; ^1^H-NMR: δ 7.81 (d, *J* = 7.6 Hz, 1H), 7.55 (d, *J* = 7.1 Hz, 1H), 7.47 (s, 1H), 7.37 (s, 1H), 3.65 (s, 2H), 2.45 (s, 3H), 2.01 (s, 3H); ESI-MS: *m*/*z* [M + H]^+^ 173.

*2-Methyl-2,3-dihydro-1H-inden-1-one* (**20a**). To a solution of **19a** (1.44 g, 10 mmol) in EtOAc (20 mL), 10% Pd/C (20% weight of compound **19a**) was added. The mixture was stirred overnight under a hydrogen atmosphere at room temperature. Insoluble materials were removed by filtration and washed with EtOAc. The filtrate was evaporated to dryness under reduced pressure to give **20a** (1.43 g, 98%) as a colorless transparent liquid. ^1^H-NMR: δ 7.76 (d, *J* = 7.7 Hz, 1H), 7.59 (d, *J* = 7.6, 1H), 7.45 (d, *J* = 7.7 Hz, 1H), 7.37 (t, *J* = 7.4 Hz, 1H), 3.45–3.36 (m, 1H), 2.79–2.68 (m, 2H), 1.35–1.30 (m, 3H); ESI-MS: *m*/*z* [M + H]^+^ 147.

*2-Isopropyl-2,3-dihydro-1H-inden-1-one* (**20b**). The title compound was prepared from **19b** in a manner similar to that described for **20a** as a colorless transparent liquid (97%). ^1^H-NMR: δ 7.75 (d, *J* = 7.7 Hz, 1H), 7.59 (td, *J* = 7.6, 1.1 Hz, 1H), 7.49 (d, *J* = 7.7 Hz, 1H), 7.39–7.34 (m, 1H), 3.16 (dd, *J* = 17.4, 8.1 Hz, 1H), 2.95 (dd, *J* = 17.4, 4.0 Hz, 1H), 2.82–2.79 (m, 1H), 2.45–2.42 (m, 1H), 1.07 (d, *J* = 6.9 Hz, 3H), 0.81 (d, *J* = 6.8 Hz, 3H); ESI-MS: *m*/*z* [M + H]^+^ 175.

*2-Methyl-6-nitro-2,3-dihydro-1H-inden-1-one* (**21a**). The title compound was prepared from **20a** in a manner similar to that described for **10** as a yellow solid (67%). m.p. 64~66 °C; ^1^H-NMR: δ 8.58 (d, *J* = 2.1 Hz, 1H), 8.46 (dd, *J* = 8.4, 2.2 Hz, 1H), 7.64 (d, *J* = 8.4 Hz, 1H), 3.55–3.50 (m, 1H), 2.91–2.79 (m, 2H), 1.37 (d, *J* = 7.3 Hz, 3H); ESI-MS: *m*/*z* [M + H]^+^ 192.

*2-Isopropyl-6-nitro-2,3-dihydro-1H-inden-1-one* (**21b**). The title compound was prepared from **20b** in a manner similar to that described for **10** as a yellow solid (73%). m.p. 72~76 °C; ^1^H-NMR: δ 8.55 (d, *J* = 2.0 Hz, 1H), 8.44 (dd, *J* = 8.4, 2.2 Hz, 1H), 7.65 (d, *J* = 8.3 Hz, 1H), 3.28 (dd, *J* = 18.3, 8.2 Hz, 1H), 3.04 (dd, *J* = 18.3, 4.1 Hz, 1H), 2.82-2.79 (m, 1H), 2.51–2.40 (m, 1H), 1.07 (d, *J* = 6.9 Hz, 3H), 0.83 (d, *J* = 6.8 Hz, 3H); ESI-MS: *m*/*z* [M + H]^+^ 192.

*2-Methyl-6-nitro-2,3-dihydro-1H-inden-1-ol* (**22a**). The title compound was prepared from **21a** in a manner similar to that described for **11** as a white solid (86%). m.p. 81~84 °C; ^1^H-NMR: δ 8.58 (d, *J* = 5.0 Hz, 1H), 8.06 (s, 1H), 7.45 (d, *J* = 8.0 Hz, 1H), 5.72 (d, *J* = 6.0 Hz, 1H), 4.62–4.59 (s, 1H), 3.10–3.05 (m, 1H), 2.58–2.53 (m, 1H), 2.24–2.14 (m, 1H), 1.20 (d, *J* = 6.7 Hz, 3H);ESI-MS: *m*/*z* [M + H]^+^ 194.

*2-Isopropyl-6-nitro-2,3-dihydro-1H-inden-1-ol* (**22b**). The title compound was prepared from **21b** in a manner similar to that described for **11** as a white solid (89%). m.p. 90~93 °C; ^1^H-NMR: δ 8.15–8.04 (m, 1H), 7.70 (m, 1H), 7.41 (m, 1H), 3.22 (d, *J* = 3.3 Hz, 1H), 3.54 (dd, *J* = 17.9, 7.3 Hz, 1H), 3.15 (dd, *J* = 17.9, 9.8 Hz, 1H), 2.05–1.92 (m, 1H), 1.55 (m, 1H), 1.14 (t, *J* = 5.5 Hz, 3H), 1.09 (d, *J* = 6.5 Hz, 3H)); ESI-MS: *m*/*z* [M + H]^+^ 222.

*2-Methyl-5-nitro-1H-indene* (**23a**). The title compound was prepared from **22a** in a manner similar to that described for **12** as a white solid (81%). m.p.: 68~72 °C; ^1^H-NMR: δ 7.76 (d, *J* = 7.6 Hz, 1H), 7.59 (t, *J* = 7.4 Hz, 1H), 7.46 (d, *J* = 7.7 Hz, 1H), 5.02 (d, *J* = 5.9 Hz, 1H), 3.41 (s, 8.7 Hz, 2H), 1.32 (d, *J* = 7.2 Hz, 3H); ESI-MS: *m*/*z* [M + H]^+^ 176.

*2-Isopropyl-5-nitro-1H-indene* (**23b**). The title compound was prepared from **22b** in a manner similar to that described for **12** as a white solid (82%). m.p. 78~81 °C; ^1^H-NMR: δ 8.07 (d, *J* = 1.8 Hz, 1H), 8.00 (dd, *J* = 8.1, 1.8 Hz, 1H), 7.46 (d, *J* = 8.1 Hz, 1H), 6.56 (s, 1H), 3.44 (s, 2H), 2.90–2.73 (m, 1H), 1.25 (s, 3H), 1.24 (s, 3H); ESI-MS: *m*/*z* [M + H]^+^ 204.

*1-Methoxy-2-methyl-6-nitro-2,3-dihydro-1H-indene* (**27a**). To a solution of **22a** (106 mg, 0.55 mmol) and trimethyl orthoformate (1 mL) in anhydrous CH_2_Cl_2_ (2 mL), bismuth trichloride (173 mg, 0.55 mmol) was added at room temperature. The mixture was stirred at room temperature for 7 h and then treated with aqueous 1 N NaHCO_3_ until basic. After adding water (50 mL), the mixture was partitioned between water and EtOAc. The organic layer was washed with a saturated aqueous solution of brine, dried over anhydrous Na_2_SO_4_, and concentrated under vacuum. The residue was purified by column chromatography on silica gel (eluent: hexane/EtOAc = 30/1) to give **27a** (27 mg, 24%) as a yellow oil. ^1^H-NMR: δ 8.22 (s, 1H), 8.15 (dd, *J* = 8.3, 2.1 Hz, 1H), 7.35 (d, *J* = 8.3 Hz, 1H), 4.42 (d, *J* = 4.4 Hz, 1H), 3.52 (s, 3H), 3.31–3.26 (m, 1H), 2.64–2.56 (m, 1H), 2.56–2.50 (m, 1H), 1.19 (d, *J* = 7.0 Hz, 3H); ESI-MS: *m*/*z* [M + H]^+^ 208.

*1-Ethoxy-2-methyl-6-nitro-2,3-dihydro-1H-indene* (**27b**). The title compound was prepared from **22b** and triethyl orthoformate in a manner similar to that described for **27a** as yellow oil (33%). ^1^H-NMR: δ 8.26 (s, 1H), 8.16 (d, *J* = 8.3 Hz, 1H), 7.38 (d, *J* = 8.1 Hz, 1H), 4.92 (d, *J* = 6.4 Hz, 1H), 3.65 (d, *J* = 7.0 Hz, 2H), 2.80–2.73 (m, 2H), 2.65 (q, *J* = 6.5, 1H), 1.33 (d, *J* = 6.6 Hz, 3H), 1.31–1.27 (m, 3H); ESI-MS: *m*/*z* [M + H]^+^ 222.

*2-Isopropyl-1-methoxy-6-nitro-2,3-dihydro-1H-indene* (**28a**). The title compound was prepared from **22a** in a manner similar to that described for **27a** as yellow oil (27%). ^1^H-NMR: δ 8.20 (d, *J* = 2.0 Hz, 1H), 8.18 (dd, *J* = 8.2, 2.2 Hz, 1H), 7.41 (d, *J* = 8.2 Hz, 1H), 4.51 (d, *J* = 3.2 Hz, 1H), 3.34 (s, 3H), 3.00–2.86 (m, 2H), 2.09–2.01 (m, 1H), 2.00–1.95 (m, 1H), 1.07 (d, *J* = 6.3 Hz, 3H), 1.00 (d, *J* = 6.6 Hz, 3H); ESI-MS: *m*/*z* [M + H]^+^ 236.

*1-Ethoxy-2-isopropyl-6-nitro-2,3-dihydro-1H-indene* (**28b**). The title compound was prepared from **22b** and triethyl orthoformate in a manner similar to that described for **27a** as yellow oil (19%). ^1^H-NMR: δ 8.25 (d, *J* = 4.4 Hz, 1H), 8.14 (dd, *J* = 8.3, 2.2 Hz, 1H), 7.35 (d, *J* = 8.3 Hz, 1H), 4.62 (d, *J* = 5.3 Hz, 1H), 3.20 (q, *J* =8.5 Hz, 2H), 2.83–2.75 (m, 2H), 2.66–2.56 (m, 1H), 2.19–2.06 (m, 1H), 1.09 (d, *J* = 6.5 Hz, 5H), 1.01–0.99 (m, 3H); ESI-MS: *m*/*z* [M + H]^+^ 250.

*Dimethyl Terephthalate* (**32**). To a solution of terephthalic acid (**31**, 6.0 g, 36.0 mmol) in methanol (150 mL), thionyl chloride (7.7 mL, 108 mmol) was added dropwise at 0 °C. The mixture was stirred at room temperature for 17 h and then saturated potassium carbonate solution was added until no bubbles were generated. The methanol was removed by distillation. After adding water (40 mL), the mixture was partitioned between water and ether. The organic layer was washed with a saturated aqueous solution of NaHCO_3_ and brine, dried over anhydrous Na_2_SO_4_, and concentrated under vacuum to give **32** (6.84 g, 98%) as a white solid. m.p. 141~143 °C (ether); ESI-MS: *m*/*z* [M + H]^+^ 195.

*4-(Methoxycarbonyl)benzoic acid* (**33**). To a solution of **32** (2.0 g, 10.0 mmol) in methanol and ether (methanol:ether = 1:1, 20 mL), a solution of KOH (0.58 g, 10.0 mmol) in methanol and water (methanol:water = 10:1, 10 mL) was added dropwise at 0 °C. The mixture was stirred at room temperature for 24 h. After adding water (50 mL), the mixture was partitioned between water and ether. Then treating the aqueous layer successively with 1 N HCl until pH = 1. The mixture was partitioned between water and EtOAc. The organic layer was washed with a saturated aqueous solution of brine, dried over anhydrous Na_2_SO_4_, and concentrated under vacuum to give **33** (1.0 g, 56%) as a white solid. m.p. 188~192 °C. 

*4-((2,3-Dihydro-1H-inden-5-yl)carbamoyl) benzoate* (**35a**). To a solution of **12** (80.5 mg, 0.5 mmol) in EtOAc (20 mL), 10% Pd/C (20% net weight of compound **19a**) was added. The mixture was stirred overnight under a hydrogen atmosphere at room temperature. Insoluble materials were removed by filtration and washed with EtOAc. The filtrate was evaporated to dryness under reduced pressure to give **13** as brown oil (65 mg, 98%). To a solution of **33** (150 mg, 0.8 mmol) in thionyl chloride (4 mL), a drop of pyridine was added and refluxed for 24 h. The thionyl chloride was removed by distillation and get **34** as pale yellow solid. **34** was used directly in the next reaction. To a solution of **13** (65 mg, 0.5 mmol) in anhydrous pyridine (2 mL), **34** was added in CH_2_Cl_2_ (2 mL) dropwise at 0 °C. The mixture was stirred at room temperature for 7 h and then the methanol was removed by distillation. The organic layer was treated successively with 1 N HCl until acidic. The mixture was partitioned between water and EtOAc. The organic layer was washed with a saturated aqueous solution of brine, dried over anhydrous Na_2_SO_4_, and concentrated under vacuum. The residue was purified by column chromatography on silica gel (eluent: hexane/EtOAc = 3/1) to give **35a** (118 mg, 80%) as a pale yellow solid. m.p. 138~142 °C; ^1^H-NMR: δ 8.15 (d, *J* = 6.9 Hz, 2H), 7.93 (d, *J* = 8.1 Hz, 2H), 7.60 (s, 1H), 7.30 (d, *J* = 8.1 Hz,1H), 7.22 (d, *J* = 7.9 Hz, 1H), 3.96 (s, 3H), 2.95–2.89 (m, 4H), 2.13–2.07 (m, 2H);ESI-MS: *m*/*z* [M + H]^+^ 296.

*Methyl 4-((3-methoxy-2,3-dihydro-1H-inden-5-yl)carbamoyl) benzoate* (**35b**). The title compound was prepared from **14a** in a manner similar to that described for **35a** as a pale yellow solid (87%). m.p. 148~151 °C; ^1^H-NMR: δ 8.15 (d, *J* = 8.3 Hz, 2H), 7.92 (d, *J* = 8.3 Hz, 2H), 7.86 (s, 1H), 7.72 (s, 1H), 7.50 (d, *J* = 7.3 Hz, 1H), 7.27 (d, *J* = 8.0 Hz, 1H), 4.83 (dd, *J* = 6.5, 4.2 Hz, 1H), 3.96 (s, 3H), 3.43 (s, 3H), 3.11–3.01 (m, 1H), 2.85–2.77 (m, 1H), 2.42–2.32 (m, 1H), 2.11–2.08 (m, 1H); ESI-MS: *m*/*z* [M + H]^+^ 326.

*Methyl 4-((3-ethoxy-2,3-dihydro-1H-inden-5-yl)carbamoyl) benzoate* (**35c**). The title compound was prepared from **14b** in a manner similar to that described for **35a** as a pale yellow solid (76%). m.p. 153~156 °C; ^1^H-NMR: δ 8.16 (d, *J* = 8.2 Hz, 2H), 7.93 (d, *J* = 8.1 Hz, 2H), 7.82 (s, 1H), 7.71 (s, 1H), 7.50 (d, *J* = 7.7 Hz, 1H), 7.24 (d, *J* = 8.0 Hz, 1H), 4.96–4.89 (m, 1H), 3.97 (s, 3H), 3.64 (q, *J* = 6.8, 2H), 3.09–3.03 (m, 1H), 2.83–2.77 (m, 1H), 2.44–2.36 (m, *J* = 6.7 Hz, 1H), 2.12–2.06 (m, 1H), 1.25 (t, *J* = 7.0 Hz, 3H); ESI-MS: *m*/*z* [M + H]^+^ 340.

*Methyl 4-((3-isopropoxy-2,3-dihydro-1H-inden-5-yl)carbamoyl) benzoate* (**35d**). The title compound was prepared from **14c** in a manner similar to that described for **35a** as a pale yellow solid (79%). m.p. 150~153 °C ; ^1^H-NMR: δ 8.16 (d, *J* = 7.7 Hz, 2H), 7.93 (d, *J* = 7.6 Hz, 2H), 7.80 (s, 1H), 7.50 (d, *J* = 7.2 Hz, 1H), 7.23 (d, *J* = 8.3 Hz, 1H), 5.01 (t, *J* = 5.9 Hz, 1H), 3.96 (s, 3H), 3.92–3.83 (m, 1H), 3.03 (s, 1H), 2.78 (dt, *J* = 15.7, 7.8 Hz, 1H), 2.44 (d, *J* = 6.2 Hz, 1H), 2.03 (dt, *J* = 13.1, 8.5 Hz, 1H), 1.25 (s, 6H); ESI-MS: *m*/*z* [M + H]^+^ 354. 

*Methyl 4-((3-isopropoxy-2,3-dihydro-1H-inden-5-yl)carbamoyl) benzoate* (**35e**). The title compound was prepared from **14d** in a manner similar to that described for **35a** as a pale yellow solid (73%). m.p. 159~160 °C; ^1^H-NMR: δ 8.16 (d, *J* = 8.3 Hz, 2H), 7.93 (d, *J* = 8.2 Hz, 2H), 7.81 (s, 1H), 7.66 (s, 1H), 7.52 (d, *J* = 7.8 Hz, 1H), 7.24 (s, 1H), 4.93–4.85 (m, 1H), 3.96 (s, 3H), 3.57 (dd, *J* = 15.4, 6.7 Hz, 2H), 3.04 (ddd, *J* = 16.8, 11.4, 7.0 Hz, 1H), 2.79 (s, 1H), 2.40 (dq, *J* = 8.2, 6.2 Hz, 1H), 2.15–1.99 (m, 1H), 1.67–1.58 (m, 2H), 1.41 (dd, *J* = 13.2, 7.5 Hz, 2H), 0.96–0.89 (m, 3H); ESI-MS: *m*/*z* [M + H]^+^ 368.

*Methyl 4-((3-isopropoxy-2,3-dihydro-1H-inden-5-yl)carbamoyl) benzoate* (**35f**). The title compound was prepared from **17a** in a manner similar to that described for **35a** as a yellow solid (66%). m.p. 134~135 °C; ^1^H-NMR: δ 8.16 (d, *J* = 8.4 Hz, 2H), 7.92 (d, *J* = 8.2 Hz, 2H), 7.75 (s, 1H), 7.63 (s, 1H), 7.31 (d, *J* = 5.7 Hz, 1H), 4.28 (t, *J* = 5.3 Hz, 1H), 3.97 (s, 3H), 3.39 (s, 3H), 3.21–3.13 (m, 2H), 3.03–2.95 (m, 2H); ESI-MS: *m*/*z* [M + H]^+^ 326.

*Methyl 4-((3-isopropoxy-2,3-dihydro-1H-inden-5-yl)carbamoyl) benzoate* (**35g**). The title compound was prepared from **17b** in a manner similar to that described for **35a** as a yellow solid (71%). m.p. 137~139 °C; ^1^H-NMR: δ 8.16 (d, *J* = 8.3 Hz, 2H), 7.92 (d, *J* = 8.3 Hz, 2H), 7.75 (s, 1H), 7.62 (s, 1H), 7.30 (d, *J* = 9.3 Hz, 1H), 7.20 (d, *J* = 7.8 Hz, 1H), 4.37 (t, *J* = 5.0 Hz, 1H), 3.56 (d, *J* = 7.0 Hz, 2H), 3.21-3.14 (m, 2H), 3.04–2.93 (m, 2H), 1.23 (t, *J* = 7.0 Hz, 3H); ESI-MS: *m*/*z* [M + H]^+^ 340.

*Methyl 4-((3-isopropoxy-2,3-dihydro-1H-inden-5-yl)carbamoyl) benzoate* (**35h**). The title compound was prepared from **23b** in a manner similar to that described for **35a** as a pale yellow solid (87%). m.p. 144~149 °C; ^1^H-NMR: δ 8.19 (d, *J* = 8.3 Hz, 2H), 7.98 (s, 1H), 8.00–7.93 (m, 2H), 7.84 (s, 1H), 7.54 (d, *J* = 8.2 Hz, 1H), 3.98 (d, *J* = 4.4 Hz, 3H), 3.21–3.11 (m, 3H), 2.79–2.70 (m, 3H), 1.25 (d, *J* = 20.0 Hz, 6H); ESI-MS: *m*/*z* [M + H]^+^ 338.

*Methyl 4-((3-isopropoxy-2,3-dihydro-1H-inden-5-yl)carbamoyl) benzoate* (**35k**). The title compound was prepared from **21a** in a manner similar to that described for **35a** as a pale yellow solid (77%). m.p. 167~171 °C; ^1^H-NMR: δ 8.17 (s, 2H), 8.11 (d, *J* = 6.4 Hz, 1H), 7.96 (s, 2H), 7.82 (s, 1H), 7.50 (d, *J* = 8.2 Hz, 1H), 3.97 (s, 3H), 3.41 (dd, *J* = 16.4, 7.3 Hz, 1H), 2.80–2.75 (m, 1H), 2.75 (s, 1H), 1.33 (d, *J* = 7.3 Hz, 3H); ESI-MS: *m*/*z* [M + H]^+^ 324.

*Methyl 4-((3-isopropoxy-2,3-dihydro-1H-inden-5-yl)carbamoyl) benzoate* (**35l**). The title compound was prepared from **21b** in a manner similar to that described for **35a** as a pale yellow solid (89%). m.p. 170~174 °C; ^1^H-NMR: δ 8.21 (d, *J* = 8.2 Hz, 1H), 8.18 (d, *J* = 8.4 Hz, 2H), 8.00 (d, *J* = 8.2 Hz, 2H), 7.86 (s, 1H), 7.53 (d, *J* = 8.3 Hz, 1H), 3.15 (dd, *J* = 17.4, 8.0 Hz, 1H), 2.94 (dd, *J* = 17.4, 3.8 Hz, 1H), 2.72 (dt, *J* = 8.1, 4.1 Hz, 1H), 2.43–2.33 (m, 1H), 1.06 (d, *J* = 6.9 Hz, 3H), 0.79 (d, *J* = 6.8 Hz, 3H; ESI-MS: *m*/*z* [M + H]^+^ 352.

*Methyl 4-((3-isopropoxy-2,3-dihydro-1H-inden-5-yl)carbamoyl) benzoate* (**35m**). The title compound was prepared from **27a** in a manner similar to that described for **35a** as a pale yellow solid (91%). m.p. 147~149 °C; ^1^H-NMR: δ 8.16 (dd, *J* = 8.2, 4.4 Hz, 2H), 7.93 (d, *J* = 7.2 Hz, 2H), 7.73 (s, 1H), 7.48 (d, *J* = 7.1 Hz, 1H), 7.23 (d, *J* = 8.2 Hz, 1H), 4.41 (d, *J* = 3.6 Hz, 1H), 3.96 (s, 3H), 3.49 (s, 3H), 3.25–3.17 (m, 1H), 2.57–2.50 (m, 1H), 2.43 (dd, *J* = 15.8, 3.2 Hz, 1H), 1.17 (d, *J* = 7.0, 3H); ESI-MS: *m*/*z* [M + H]^+^ 340.

*Methyl 4-((3-ethoxy-2-methyl-2, 3-dihydro-1H-inden-5-yl)carbamoyl) benzoate* (**35n**). The title compound was prepared from **27b** in a manner similar to that described for **35a** as a pale yellow solid (86%). m.p. 140~142 °C; ^1^H-NMR: δ 8.16 (d, *J* = 8.2 Hz, 2H), 7.93 (d, *J* = 8.1 Hz, 2H), 7.82 (s, 1H), 7.70 (s, 1H), 7.46 (d, *J* = 7.4 Hz, 1H), 7.21 (d, *J* = 8.0 Hz, 1H), 4.49 (d, *J* = 4.6 Hz, 1H), 3.97 (s, 3H), 3.71 (q, *J* = 7.0 Hz, 2H), 3.19 (dd, *J* = 15.7, 7.6 Hz, 1H), 2.57–2.45 (m, 1H), 2.42 (dd, *J* = 15.6, 5.8 Hz, 1H), 1.27 (t, *J* = 6.9 Hz, 3H), 1.18 (d, *J* = 7.0 Hz, 3H); ESI-MS: *m*/*z* [M + H]^+^ 354.

*4-((2-Isopropyl-3-methoxy-2,3-dihydro-1H-inden-5-yl)carbamoyl) benzoate* (**35o**). The title compound was prepared from **28a** in a manner similar to that described for **35a** as a pale yellow solid (87%). m.p. 152~155 °C; ^1^H-NMR: δ 8.16 (d, *J* = 8.3 Hz, 2H), 7.94 (d, *J* = 8.3 Hz, 2H), 7.89 (s, 1H), 7.72 (s, 1H), 7.48 (d, *J* = 7.8 Hz, 1H), 7.22 (d, *J* = 8.1 Hz, 1H), 4.70 (d, *J* = 5.0 Hz, 1H), 3.97 (s, 3H), 3.48 (s, 3H), 3.08 (dd, *J* = 16.2, 8.4 Hz, 1H), 2.60 (dd, *J* = 16.2, 6.3 Hz, 1H), 2.31 (tt, *J* = 8.4, 6.4 Hz, 1H), 1.84 (dq, *J* = 13.4, 6.7 Hz, 1H), 1.00 (d, *J* = 6.8 Hz, 3H), 0.94 (t, *J* = 7.4 Hz, 3H); ESI-MS: *m*/*z* [M + H]^+^ 368.

*Methyl 4-((3-ethoxy-2-isopropyl-2,3-dihydro-1H-inden-5-yl)carbamoyl) benzoate* (**35p**). The title compound was prepared from **28b** in a manner similar to that described for **35a** as a pale yellow solid (97%). m.p. 146~149 °C; ^1^H-NMR: δ 8.15 (s, 2H), 7.93 (d, *J* = 8.2 Hz, 2H), 7.74 (s, 1H), 7.55 (s, 1H), 7.17 (d, *J* = 7.9 Hz, 1H), 4.56 (d, *J* = 5.0 Hz, 1H), 3.96 (s, 3H), 3.00 (q, *J* =8.0 Hz, 2H), 2.68–2.58 (m, 2H), 2.21–2.16 (m, 1H), 1.72–1.63 (m, 1H), 1.02 (d, *J* = 6.8 Hz, 3H), 0.98 (d, *J* = 6.6 Hz, 6H); ESI-MS: *m*/*z* [M + H]^+^ 368.

*4-((2,3-Dihydro-1H-inden-5-yl)carbamoyl) benzoate* (**35i**). To a solution of **23a** (175 mg, 1.0 mmol) and Fe (392 mg, 7.0 mmol) in EtOH (20 mL), AcOH (0.8 mL) was added. The mixture was refluxing for 2 h under a nitrogen atmosphere at room temperature. Insoluble materials were removed by filtration and washed with EtOAc. The filtrate was evaporated to dryness under reduced pressure. The mixture was partitioned between water and EtOAc. The organic layer was washed with a saturated aqueous solution of brine, dried over anhydrous Na_2_SO_4_, and concentrated under vacuum. The residue **25a** was used directly in the next reaction. To a solution of **33** (150 mg, 0.8 mmol) in thionyl chloride (4 mL), a drop of pyridine was added and refluxed for 24 h. The thionyl chloride was removed by distillation and get **34** as pale yellow solid. To a solution of **25a** (45 mg, 0.3 mmol) in anhydrous pyridine (2 mL), **34** was added in CH_2_Cl_2_ (2 mL) dropwise at 0 °C. The mixture was stirred at room temperature for 7 h and then the methanol was removed by distillation. The organic layer was neutralized with 1 N HCl. The mixture was partitioned between water and EtOAc. The organic layer was washed with a saturated aqueous solution of brine, dried over anhydrous Na_2_SO_4_, and concentrated under vacuum. The residue was purified by column chromatography on silica gel (eluent: hexane/EtOAc = 3/1) to give **35i** (77 mg, 84%) as a pale yellow solid. m.p. 157~159 °C; ^1^H-NMR: δ 8.15 (d, *J* = 8.4 Hz, 2H), 7.94 (d, *J* = 8.2 Hz, 2H), 7.83 (s, 1H), 7.62 (s, 1H), 7.34 (d, *J* = 7.9 Hz, 1H), 6.48 (s, 1H), 3.96 (s, 3H), 3.29 (s, 2H), 2.17 (s, 3H); ESI-MS: *m*/*z* [M + H]^+^ 338.

*Methyl 4-((2-isopropyl-1H-inden-5-yl)carbamoyl) benzoate* (**35j**). The title compound was prepared from **23b** in a manner similar to that described for **35i** as a pale yellow solid (67%). m.p. 155~156 °C; ^1^H-NMR: δ 8.16 (d, *J* = 8.4 Hz, 2H), 7.94 (d, *J* = 8.3 Hz, 2H), 7.78 (s, 1H), 7.63 (s, 1H), 7.35 (s, 1H), 6.50 (s, 1H), 3.97 (s, 3H), 3.34 (s, 2H), 2.81–2.76 (m, 1H), 1.24 (d, *J* = 6.8, 3H); ESI-MS: *m*/*z* [M + H]^+^ 336.

*4-((2,3-Dihydro-1H-inden-5-yl)carbamoyl)benzoic acid* (**36a**). To a solution of **35a** (56 mg, 0.2 mmol) in MeOH (2 mL), 0.5 N LiOH (0.4 mL) was added dropwise at 0 °C. The mixture was stirred at room temperature for 48 h and then neutralized with 1 N HCl. The MeOH was removed by distillation. After adding water (10 mL), the mixture was partitioned between water and EtOAc. The organic layer was washed with a saturated aqueous solution of brine, dried over anhydrous Na_2_SO_4_, and concentrated under vacuum. The residue was purified by column chromatography on silica gel (eluent: hexane/EtOAc/AcOH = 30/10/1) to give **36a** (52 mg, 92%) as a white solid, purity: 97%. m.p. >250 °C; ^1^H-NMR: δ 13.26 (s, 1H), 10.30 (s, 1H), 8.14–7.92 (m, 4H), 7.68 (s, 1H), 7.47 (d, *J* = 8.0 Hz, 1H), 7.19 (d, *J* = 8.1 Hz, 1H), 2.93–2.76 (m, 4H), 2.07–1.97 (m, 2H); ESI-MS: *m*/*z* [M − H]^−^ 280.

*4-((3-Methoxy-2,3-dihydro-1H-inden-5-yl)carbamoyl)benzoic acid* (**36b**). The title compound was prepared from **35b** in a manner similar to that described for **36a** as a white solid (91%), purity: 96%. m.p. >250 °C; ^1^H-NMR: δ 10.38 (s, 1H), 8.15–8.01 (m, 4H), 7.86 (s, 1H), 7.63 (d, *J* = 8.1 Hz, 1H), 7.25 (d, *J* = 8.2 Hz, 1H), 4.86–4.72 (m, 1H), 3.33(s, 3H),3.00–2.87 (m, 1H), 2.81–2.69 (m, 1H), 2.36–2.32 (m, 1H), 1.99–1.93 (m, 1H); ESI-MS: *m*/*z* [M − H]^−^ 310. HRMS (ESI) calcd [M + H]^+^ for C_18_H_18_NO_4_ 312.1230, found 312.1234.

*4-((3-Methoxy-2,3-dihydro-1H-inden-5-yl)carbamoyl)benzoic acid* (**36c**). The title compound was prepared from **35c** in a manner similar to that described for **36a** as a white solid (99%), purity: 95%. m.p. >250 °C; ^1^H-NMR: δ 10.37 (s, 1H), 8.07–8.03 (m, 4H), 7.81 (s, 1H), 7.63 (dd, *J* = 8.0, 2.0 Hz, 1H), 7.23 (d, *J* = 8.2 Hz, 1H), 4.89–4.83 (m, 1H), 3.60–3.50 (m, 2H), 2.94–2.88 (m, 1H), 2.76–2.69 (m, 1H), 2.36–2.30 (m, 1H), 1.95–1.89 (m, 1H), 1.15 (t, *J* = 7.0 Hz, 3H); ESI-MS: *m*/*z* [M − H]^−^ 324. HRMS (ESI) calcd [M + H]^+^ for C_19_H_20_NO_4_ 326.1387, found 326.1392.

*4-((3-Isopropoxy-2,3-dihydro-1H-inden-5-yl)carbamoyl)benzoic acid* (**36d**). The title compound was prepared from **35d** in a manner similar to that described for **36a** as a white solid (97%), purity: 97%. m.p. >250 °C; ^1^H-NMR: δ 13.24 (s, 1H), 10.36 (s, 1H), 8.05 (s, 4H), 7.74 (s, 1H), 7.64 (dd, *J* = 8.1, 1.9 Hz, 1H), 7.21 (d, *J* = 8.2 Hz, 1H), 4.96 (t, *J* = 6.1 Hz, 1H), 3.93–3.74 (m, 2H), 3.85–3.79 (m, 1H), 2.94–2.84 (m, 1H), 2.73–2.67 (m, 1H), 2.40–2.34 (m, 1H), 1.87–1.81 (m, 1H), 1.17 (d, *J* = 6.0, 3H) , 1.16 (d, *J* = 6.5, 3H); ESI-MS: *m*/*z* [M − H]^−^ 338. HRMS (ESI) calcd [M + H]^+^ for C_20_H_22_NO_4_ 340.1543, found 340.1550.

*4-((3-Butoxy-2,3-dihydro-1H-inden-5-yl)carbamoyl)benzoic acid* (**36e**). The title compound was prepared from **35e** in a manner similar to that described for **36a** as a white solid (90%). purity: 96%. m.p. >250 °C; ^1^H-NMR: δ 13.26 (s, 1H), 10.37 (s, 1H), 8.05 (d, *J* = 2.1 Hz, 4H), 7.79 (s, 1H), 7.63 (dd, *J* = 8.0 Hz, 1H), 7.21 (d, *J* = 8.0 Hz, 1H), 4.89–4.80 (m, 1H), 3.51 (td, *J* = 6.5, 2.5 Hz, 2H), 2.98–2.85 (m, 1H), 2.78–2.66 (m, 1H), 2.38–2.30 (m, 1H), 1.94–1.86 (m, 1H), 1.56–1.42 (m, 2H), 1.39–1.31 (m, 2H), 0.88 (t, *J* = 7.4 Hz, 3H); ESI-MS: *m*/*z* [M − H]^−^ 352. HRMS (ESI) calcd [M + H]^+^ for C_21_H_24_NO_4_ 354.1700, found 354.1703.

*4-((2-Methoxy-2,3-dihydro-1H-inden-5-yl)carbamoyl)benzoic acid* (**36f**). The title compound was prepared from **35f** in a manner similar to that described for **36a** as a white solid (86%), purity: 98%. m.p. >250 °C; ^1^H-NMR: δ 8.15 (d, *J* = 8.3 Hz, 2H), 7.92 (d, *J* = 8.2 Hz, 2H), 7.80 (s, 1H), 7.62 (s, 1H), 7.31 (d, *J* = 7.8 Hz, 1H), 7.21 (d, *J* = 8.1 Hz, 1H), 4.31–4.23 (m, 1H), 3.96 (s, 3H), 3.38 (s, 3H), 3.20–3.12 (m, 2H), 3.03-2.94 (m, 2H); ESI-MS: *m*/*z* [M − H]^−^ 310. HRMS (ESI) calcd [M + H]^+^ for C_18_H_18_NO_4_ 312.1230, found 312.1240.

*4-((2-Ethoxy-2,3-dihydro-1H-inden-5-yl)carbamoyl)benzoic acid* (**36g**). The title compound was prepared from **35g** in a manner similar to that described for **36a** as a white solid (97%), purity: 97%. m.p. >250 °C; ^1^H-NMR: δ 10.37 (s, 1H), 8.12–8.01 (m, 4H), 7.82 (s, 1H), 7.65 (dd, *J* = 8.1, 1.7 Hz, 1H), 7.24 (d, *J* = 8.2 Hz, 1H), 4.94–4.80 (m, 1H), 3.62–3.54 (m, 2H), 2.97–2.88 (m, 1H), 2.78–2.69 (m, 1H), 2.37–2.31 (m, 1H), 1.98–1.90 (m, 1H), 1.16 (t, *J* = 7.0 Hz, 3H); ESI-MS: *m*/*z* [M − H]^−^ 324. HRMS (ESI) calcd [M + H]^+^ for C_19_H_20_NO_4_ 326.1387, found 326.1396.

*4-((2-Isopropyl-2,3-dihydro-1H-inden-5-yl)carbamoyl)benzoic acid* (**36h**). The title compound was prepared from **35h** in a manner similar to that described for **36a** as a white solid (94%), purity: 96%. m.p. >250 °C; ^1^H-NMR: δ 8.07–8.02 (m, 4H), 7.63 (s, 1H), 7.47 (d, *J* = 7.9 Hz, 1H), 7.15 (d, *J* = 8.1 Hz, 1H), 2.98–2.90 (m, 2H), 2.66–2.52 (m, 2H), 2.15–2.10 (m, 1H), 1.68–1.10 (m, 1H), 0.95 (d, *J* = 6.5 Hz, 3H), 0.94 (d, *J* = 6.5 Hz, 3H); ESI-MS: *m*/*z* [M − H]^−^ 322. HRMS (ESI) calcd [M + H]^+^ for C_20_H_22_NO_3_ 324.1594, found 324.1600.

*4-((2-Methyl-1H-inden-5-yl)carbamoyl)benzoic acid* (**36i**). The title compound was prepared from **35i** in a manner similar to that described for **36a** as a white solid (98%), purity: 95%. m.p. >250 °C; ^1^H-NMR: 10.31 (s, 1H), 8.07–8.02 (m, 4H), 7.78 (s, 1H), 7.48 (d, *J* = 6.4 Hz, 1H), 7.26 (d, *J* = 8.1 Hz, 1H), 6.51 (s, 1H), 3.29 (s, 2H), 2.12 (s, 3H); ESI-MS: *m*/*z* [M − H]^−^ 292. HRMS (ESI) calcd [M + H]^+^ for C_18_H_16_NO_3_ 294.1125, found 294.1129.

*4-((2-Isopropyl-1H-inden-5-yl)carbamoyl)benzoic acid* (**36j**). The title compound was prepared from **35j** in a manner similar to that described for **36a** as a white solid (83%), purity: 95%. m.p. >250 °C; ^1^H-NMR: δ 13.25 (s, 1H), 10.34 (s, 1H), 8.09–8.06 (m, 4H), 7.72 (s, 1H), 7.47 (dd, *J* = 8.0, 1.5 Hz, 1H), 7.35 (d, *J* = 8.1 Hz, 1H), 6.53 (s, 1H), 3.37 (s, 2H), 2.79–2.72 (m, 1H), 1.20 (d, *J* = 6.5, 3H), 1.19 (d, *J* = 6.5, 3H); ESI-MS: *m*/*z* [M − H]^−^ 320. HRMS (ESI) calcd [M + H]^+^ for C_20_H_20_NO_3_ 322.1438, found 322.1440.

*4-((2-Methyl-3-oxo-2,3-dihydro-1H-inden-5-yl)carbamoyl)benzoic acid* (**36k**). The title compound was prepared from **35k** in a manner similar to that described for **36a** as a white solid (92%), purity: 95%. m.p. >250 °C (AcOH–EtOAc–hexane); ^1^H-NMR: δ 10.60 (s, 1H), 8.09–8.04 (m, 4H), 8.07 (d, *J* = 2.8 Hz, 1H), 8.00 (dd, *J* = 8.3, 2.1 Hz, 1H), 7.56 (d, *J* = 8.4 Hz, 1H), 3.38–3.36 (m, *J* = 7.4 Hz, 1H), 2.78–2.72 (m, 1H), 2.70–2.65 (m, 1H), 1.20 (d, *J* = 7.4 Hz, 3H); ESI-MS: *m*/*z* [M − H]^−^ 308. HRMS (ESI) calcd. [M + H]^+^ for C_18_H_16_NO_4_ 310.1074, found 310.1069.

*4-((2-Isopropyl-3-oxo-2,3-dihydro-1H-inden-5-yl)carbamoyl)benzoic acid* (**36l**). The title compound was prepared from **35l** in a manner similar to that described for **36a** as a white solid (97%), purity: 98%. m.p. >250 °C; ^1^H-NMR: δ 13.30 (s, 1H), 10.61 (s, 1H), 8.14 (d, *J* = 1.8 Hz, 1H), 8.08 (d, *J* = 3.9 Hz, 4H), 8.00 (dd, *J* = 8.3, 2.0 Hz, 1H), 7.60 (d, *J* = 8.3 Hz, 1H), 3.13 (dd, *J* = 17.4, 8.0 Hz, 1H), 2.88 (dd, *J* = 17.5, 3.8 Hz, 1H), 2.73 (dt, *J* = 8.0, 4.1 Hz, 1H), 2.30–2.24 (m, 1H), 1.01 (d, *J* = 6.9 Hz, 3H), 0.74 (d, *J* = 6.8 Hz, 3H); ESI-MS: *m*/*z* [M − H]^−^ 336. HRMS (ESI) calcd [M + H]^+^ for C_20_H_20_NO_4_ 338.1387, found 338.1384.

*4-((3-Methoxy-2-methyl-2,3-dihydro-1H-inden-5-yl)carbamoyl)benzoic acid* (**36m**). The title compound was prepared from **35m** in a manner similar to that described for **36a** as a white solid (89%), purity: 99%. m.p. >250 °C (AcOH–EtOAc–hexane); ^1^H-NMR: δ 13.26 (s, 1H), 10.36 (s, 1H), 8.06–8.04 (m, 4H), 7.84 (s, 1H), 7.63 (dd, *J* = 8.1, 1.8 Hz, 1H), 7.23 (d, *J* = 8.0, 1H), 4.37 (d, *J* = 4.3 Hz, 1H), 3.40 (s, 3H), 3.12–3.07 (m, 1H), 2.45–2.34 (m, 2H), 1.12 (d, *J* = 6.8 Hz, 3H); ESI-MS: *m*/*z* [M − H]^−^ 324. HRMS (ESI) calcd [M + H]^+^ for C_19_H_20_NO_4_ 326.1387, found 326.1392.

*4-((3-Ethoxy-2-methyl-2,3-dihydro-1H-inden-5-yl)carbamoyl)benzoic acid* (**36n**). The title compound was prepared from **35n** in a manner similar to that described for **36a** as a white solid (97%), purity: 98%. m.p. >250 °C; ^1^H-NMR: δ 13.25 (s, 1H), 10.36 (s, 1H), 8.13–8.00 (m, 4H), 7.80 (s, 1H), 7.64 (d, *J* = 8.1 Hz, 1H), 7.21 (d, *J* = 8.1 Hz, 1H), 4.44 (d, *J* = 4.5 Hz, 1H), 3.68 (q, *J* = 7.0 Hz, 2H), 3.10–3.05 (m, 1H), 2.40–3.34 (m, 2H), 1.18 (t, *J* = 7.0 Hz, 3H), 1.13 (d, *J* = 6.6 Hz, 3H); ESI-MS: *m*/*z* [M − H]^−^ 338. HRMS (ESI) calcd [M + H]^+^ for C_20_H_22_NO_4_ 340.1543, found 340.1549.

*4-((2-Isopropyl-3-methoxy-2,3-dihydro-1H-inden-5-yl)carbamoyl)benzoic acid* (**36o**). The title compound was prepared from **35o** in a manner similar to that described for **36a** as a white solid (98%), purity: 97%. m.p. >250 °C; ^1^H-NMR: δ 13.21 (s, 1H), 10.34 (s, 1H), 8.10–8.02 (m, 4H), 7.84 (s, 1H), 7.64 (dd, *J* = 8.5, 1.5 Hz , 1H), 7.20 (d, *J* = 8.2 Hz, 1H), 4.65 (d, *J* = 3.3 Hz, 1H), 3.39 (s, 3H), 3.01–2.96 (m, 1H), 2.55–2.53 (m, 1H), 2.23–2.15 (m, 1H), 1.83–1.76 (m, 1H), 0.95 (d, *J* = 6.7 Hz, 3H), 0.90 (d, *J* = 6.7 Hz, 3H); ESI-MS: *m*/*z* [M − H]^−^ 352. HRMS (ESI) calcd [M + H]^+^ for C_21_H_24_NO_4_ 354.1700, found 354.1704.

*4-((3-Ethoxy-2-isopropyl-2,3-dihydro-1H-inden-5-yl)carbamoyl)benzoic acid* (**36p**). The title compound was prepared from **35p** in a manner similar to that described for **36a** as a white solid (98%), purity: 98%. m.p. >250 °C; ^1^H-NMR: δ 13.28 (s, 1H), 10.36 (s, 1H), 8.11–7.99 (m, 4H), 7.80 (s, 1H), 7.66–7.58 (dd, *J* = 8.0, 2.0 Hz, 1H), 7.18 (d, *J* = 8.2 Hz, 1H), 4.69 (d, *J* = 5.6 Hz, 1H), 3.72–3.57 (m, 2H), 3.33–3.31 (m, 1H), 2.94 (q, *J* = 8.4 Hz, 1H), 2.18–2.12 (m, 1H), 1.81–1.76 (m, 1H), 1.16 (t, *J* = 7.0 Hz, 3H), 0.95 (d, *J* = 6.7 Hz, 3H), 0.89 (d, *J* = 6.7 Hz, 3H); ESI-MS: *m*/*z* [M − H]^−^ 366. HRMS (ESI) calcd [M + H]^+^ for C_22_H_26_NO_4_ 368.1856, found 368.1863.

### 4.3. Biology

#### 4.3.1. Receptor Binding Assay

All synthesized compounds were tested for their binding affinity by using time resolved fluorescence resonance energy transfer (TR-FRET) assay, which used a LanthaScreen^®^ TR-FRET RAR alpha Coactivator Assay Kit (Invitrogen, Carlsbad, CA, USA). Briefly, all experiments were performed in black 384-well low-volume plates (Corning Inc., Corning, NY, USA) in dark at room temperature. The final assay volume was 20 μL. All dilutions were made in assay buffer (TR-FRET Coregulator Buffer D). The final DMSO concentration was 1%. A mixture of 5 nM RAR alpha LBD-GST, 5 nM TbAnti-GST antibody, 50 nM Fluorescein-D22 was added to the wells. The only variable is the agonist concentration (6.1 × 10^−11^~1.0 × 10^−6^ M final concentrations of each retinoid). The mixture was incubated for one hour in dark followed by fluorescence intensity determination on a SpectraMax M5 microplate reader (Molecular Devices Corporation, Sunnyvale, CA, USA) with 340 nm and 520 as excitation and emission wavelengths for terbium and 340 and 495 for fluorescein, respectively. Data were analyzed by using GraphPad Prism software (GraphPad Software, Inc., La Jolla, CA, USA) and FRET signal was determined for all treatments by dividing 520 nm/495 nm signals. Graphs plotted as fold change of FRET signal for compounds treatment over DMSO only treatment.

#### 4.3.2. Inhibition of Cell Proliferation Assay

Cells were seeded in 96-well plates (Corning Inc.) with a density of 2500 cells (NB4 or HL60) per well for overnight. Then, cells were exposed to each of the test compounds **36a**–**36p** in gradient concentration of 10^−9^, 10^−8^, 10^−7^, 10^−6^, 10^−5^ mol/L. Control cultures were treated with the same volume of DMSO. After 72 hours of incubation, the cell density in each well were fixed by trichloroacetic acid and then measured using the SRB (sulforhodamine B) method. After rinsing, the SRB was solubilized in TrisHCl, and the optical density of each culture was determined with a Bio-Tek Elx 800 absorbance microplate reader (BioTek, Shoreline, WA, USA). The OD of the treated cultures was divided by that of the control cultures treated with solvent alone.

#### 4.3.3. Cell Differentiation Assay

Cells (1 × 10^6^/mL) were treated with compounds at different concentrations based on IC_50_ in Table 2 for how long, temperature (provide the cell culture condition). After the treatment, cells were washed twice with PBS and then fixed with 75% alcohol overnight at −20 °C. The fixed cells were washed with PBS and blocked with 95 μL 3% BSA in PBS for 45 min at room temperature. The cells were incubated with 5 μL CD11b-PE at 4 °C for 45 min with protection from light. The antigens were then determined by a FACSCalibur flow cytometer (BD Biosciences Pharmingen, San Diego, CA, USA). The percentages of positive cells were quantitated using CellQuest Pro software. Cells stained with mouse IgG-PE served as negative controls. Both CD11b-PE and mouse IgG-PE antibodies were purchased from BD Biosciences. At least 10,000 cells were analyzed for each data point.

## 5. Conclusions

In summary, a series of mono-/di- substituted indene derivatives were designed and synthesized to explore the impact of the size of the hydrophobic region of ATRA derivatives on the bioactivity of related compounds. Binding, antiproliferative and cell differentiation assays showed that most of these compounds retained moderate RARα agonist activity and promising cell proliferation inhibitory activity. In particular, compound **36d** with a high RARα binding affinity exhibited a strong ability to inhibit cell proliferation and to induce differentiation in NB4 cells. Structure and activity relationship study indicates that 2-alkylindene, 2-alkylindanone, or 1-alkoxyl-2-alkylindane are generally promising structural features for potent RARα agonists. All these results taken together demonstrate that indene as a promising start point for the development of novel RARα agonists.

## Figures and Tables

**Table 1:**
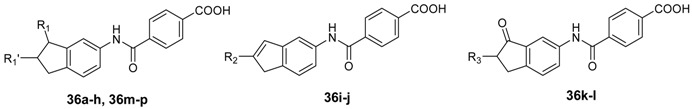
RARα binding affinity of compounds **36a**–**36p**.

Compounds	R_1_	R_1_’	R_2_	R_3_	EC_50_ (nM)
**AM80**	-	-	-	-	0.170
**36a**	H	H	-	-	12.13
**36b**	-OCH_3_	H	-	-	129.6
**36c**	-OCH_2_CH_3_	H	-	-	311.5
**36d**	-OCH(CH_3_)_2_	H	-	-	24.25
**36e**	-O(CH_2_)_3_CH_3_	H	-	-	14.88
**36f**	H	-OCH_3_	-	-	>1000
**36g**	H	-OCH_2_CH_3_	-	-	>1000
**36h**	H	-CH(CH_3_)_2_	-	-	42.96
**36i**	-	-	-CH_3_	-	14.08
**36j**	-	-	-CH(CH_3_)_2_	-	3.529
**36k**	-	-	-	-CH_3_	1375
**36l**	-	-	-	-CH(CH_3_)_2_	4.677
**36m**	-OCH_3_	-CH_3_	-	-	291.9
**36n**	-OCH_2_CH_3_	-CH_3_	-	-	146.1
**36o**	-OCH_3_	-CH(CH_3_)_2_	-	-	3.933
**36p**	-OCH_2_CH_3_	-CH(CH_3_)_2_	-	-	9.031

**Table 2:**
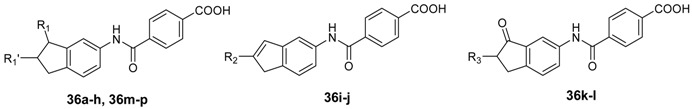
Results of cell proliferation inhibitory assay using HL60 and NB4 cells.

Compounds	R_1_	R_1_’	R_2_	R_3_	IC_50_ (μM)	
					HL60	NB4
**AM80**	-	-	-	-	0.170	13.28
**36a**	H	H	-	-	>50	6.97
**36b**	-OCH_3_	H	-	-	0.13	35.43
**36c**	-OCH_2_CH_3_	H	-	-	0.25	>50
**36d**	-OCH(CH_3_)_2_	H	-	-	>50	1.86
**36e**	-O(CH_2_)_3_CH_3_	H	-	-	>50	4.09
**36f**	H	-OCH_3_	-	-	>50	>50
**36g**	H	-OCH_2_CH_3_	-	-	>50	21.99
**36h**	H	-CH(CH_3_)_2_	-	-	0.91	2.61
**36i**	-	-	-CH_3_	-	>50	>50
**36j**	-	-	-CH(CH_3_)_2_	-	2.37	>50
**36k**	-	-	-	-CH_3_	2.66	>50
**36l**	-	-	-	-CH(CH_3_)_2_	2.11	36.90
**36m**	-OCH_3_	-CH_3_	-	-	0.43	>50
**36n**	-OCH_2_CH_3_	-CH_3_	-	-	0.80	8.51
**36o**	-OCH_3_	-CH(CH_3_)_2_	-	-	2.35	>50
**36p**	-OCH_2_CH_3_	-CH(CH_3_)_2_	-	-	1.13	7.56

**Table 3:**
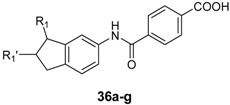
Cell differentiation potential of compounds **36a**–**g**.

Compounds	R_1_	R_1_’	CD11b (%) ^a^	
			HL60	NB4
**AM80**	-	-	4.59 (10 μM)	94.93 (10 μM)
**36a**	H	H	1.13 (50 μM)	0.97 (5 μM)
**36b**	-OCH_3_	H	nd	nd
**36c**	-OCH_2_CH_3_	H	nd	nd
**36d**	-OCH(CH_3_)_2_	H	3.77 (50 μM)	68.88 (5 μM)
**36e**	-O(CH_2_)_3_CH_3_	H	2.87 (50 μM)	15.42 (5 μM)
**36f**	H	-OCH_3_	nd	nd
**36g**	H	-OCH_2_CH_3_	5.82 (50 μM)	0.49 (20 μM)

^a^ nd: not detected.
